# Work Ability and Employment in Rheumatoid Arthritis: A Cross-Sectional Study on the Role of Muscle Strength and Lower Extremity Function

**DOI:** 10.1155/2018/3756207

**Published:** 2018-08-01

**Authors:** Carolin Berner, Sandra Haider, Igor Grabovac, Thomas Lamprecht, Karl Heinrich Fenzl, Ludwig Erlacher, Michael Quittan, Thomas Ernst Dorner

**Affiliations:** ^1^Kaiser Franz Josef Hospital, Sozialmedizinisches Zentrum-Süd, Department of Rheumatology and Osteology, Vienna, Austria; ^2^Karl Landsteiner Institute for Autoimmune Diseases and Rheumatology, Vienna, Austria; ^3^Department of Social and Preventive Medicine, Centre for Public Health, Medical University of Vienna, Vienna, Austria; ^4^Karl Landsteiner Institute for Remobilisation and Functional Health, Vienna, Austria

## Abstract

**Objective:**

The aim of the present study was to assess the association between muscle strength, lower extremity function, employment status, and work ability in RA patients.

**Methods:**

One hundred seropositive RA outpatients of working age were included in this cross-sectional study. Employment status was assessed by interview and work ability by the Work Ability Index-Single Item Scale (WAS). Muscle strength was determined using dynamometer measurement of isometric hand grip and knee extensor strength. Lower extremity function was measured using the short physical performance battery (SPPB). Regression models estimate the association between unemployment, work ability and muscle strength, and lower extremity function, controlling for sociodemographic and disease-related factors.

**Results:**

Forty-one percent of the RA patients were not gainfully employed, and their median work ability had a good WAS value (7.00 [4.00-7.00]). Patients with better knee extensor strength (OR=1.07, 95% CI [1.02-1.12) and better physical performance (OR=1.71, 95% CI [1.18-2.49]) had a significantly better chance of gainful employment. The odds for hand grip strength remained significant when adjusted for sociodemographic (OR=1.5, 95% CI [1.00-1.09]), but not for disease-specific variables. Better hand grip strength (*β*=0.25,* p*=0.039) and better knee extensor strength (*β*=0.45,* p*=0.001) as well as better lower extremity function (SPPB) (*β*=0.51,* p*<0.001) remained significantly associated with work ability following adjustment for sociodemographic and disease-specific variables.

**Conclusions:**

The association of employment status and work ability with parameters of physical fitness suggests that improvement in muscle strength and lower extremity function may positively influence work ability and employment in individuals with RA.

## 1. Background

Work disability is a major burden of rheumatic conditions [1, 2], and a substantial amount of rheumatoid arthritis- (RA-) associated work disability occurs early in the course of the disease [3]. As a result of novel therapeutic concepts, RA-induced work disability rates appear to have decreased [4, 5]; nevertheless, the risk of work disability and unemployment is still high in RA patients [5, 6], with disability rates of 20% to 30% in the first 2 to 3 years of the disease [7]. Return to work following sick leave or unemployment remains a challenge [5, 6, 8]; thus, prevention of work disability is of great importance. Even greater than treatment costs are work disability and unemployment, which pose an economic burden to both patients and society [6], making work ability and employment crucial outcomes in RA.

Work ability in RA appears to be multifactorial. A number of sociodemographic and work-related factors, such as age or type of work, have been found to be associated with work disability or unemployment in both cross-sectional and longitudinal studies [9]. Moreover, disease-related variables, including symptoms such as pain, swelling, joint stiffness [10], and functional disability [11] appear to play a major role.

Impaired lower extremity function and hand grip strength affect the quality of life of RA patients, and muscle strength is an indicator of functional disability [12–14]. Body composition, particularly a reduced amount of lean mass in the arms and legs, is associated with disability in RA patients, and weakness in RA as a result of disuse, muscle atrophy, and increased muscle mass loss is well-known [15, 16]. This condition is referred to as rheumatoid cachexia and has been reported in two-thirds of all RA patients [17, 18]. Particularly the knee extensor plays an important role in mastering activities of daily living [14] and isotonic and isometric hand exercises in RA patients can decrease pain and disease activity and increase muscle strength and function, in addition to quality of life [12].

A number of studies have identified poor physical function, with or without pain, as a significant determinant of work disability in RA [19, 20]. In healthy individuals, only a weak association could be found between muscle strength and work ability [21]; however, to the best of our knowledge, little is known regarding the association between muscle strength, lower extremity function, and work ability in RA patients.

The purpose of the present study was to assess employment status and work ability in patients of working age with RA and to explore the association between muscle strength, lower extremity function, and employment status and work ability, when controlled for relevant sociodemographic and disease-related factors.

## 2. Methods

This monocentric cross-sectional study was performed at the rheumatology outpatient clinic of the SMZ Süd Hospital (Vienna, Austria), from November 2015 to August 2016 [22]. This study was approved by the Medical Ethical Committee of the Gemeinde-Wien (EK 15-173-0915) and complied with the Declaration of Helsinki. All patients gave informed consent. Anonymity and confidentiality were guaranteed at all times. This study was registered at ClinicalTrials.gov (NCT02581852).

### 2.1. Study Sample

Participants were recruited at the rheumatology outpatient clinic while waiting for their appointment. They were considered eligible if they matched the following inclusion criteria: were of working age (≥18 and ≤65 years); had RA according to the 2010 European League Against Rheumatism (EULAR) classification for seropositive RA [23]; had sufficient knowledge of German, English, Serbo-Croatian, or Turkish to fill in the questionnaire. Patients matching the following criteria were excluded: refused to sign the informed consent; had severe comorbidities (cancer, severe cardiovascular illness, and mental illness).

### 2.2. Measures

*Employment status *was assessed by asking patients about their current employment situation. Patients were considered gainfully employed if they were in paid work at the time of study inclusion.

*Work ability *was measured using the Work Ability Index-Single Item Scale (WAS), which is the first item of the Work Ability Index (WAI). On a response scale of 0-10, where 0 represents ‘unable to work' and 10 represents ‘work ability at its best', patients reported their current estimate of work ability compared to lifetime best. The complete WAI could only be assessed for employed patients, since most items of the self-evaluation have reference to the current work setting and include the individual's current job demand (predominantly physical, mental or mixed). The WAI is commonly used to assess work ability with an adequate test-retest reliability [24, 25]. Previous research has demonstrated a high correlation between the WAI and WAS and has established the WAS as a reliable measure for assessing the status and progress of work ability [26, 27]. As proposed by Gould et al. [28], classification of the WAS is according to the WAI as follows: poor (0-5 points), moderate (6-7 points), good (8-9 points), and excellent (10 points) work ability.

### 2.3. Independent Variables and Covariates

*Sociodemographic and disease-related factors/characteristics* were assessed by clinical examination and an interview-based questionnaire. Sociodemographic characteristics were gender, age, marital status, and highest level of education. Disease-related characteristics included disease duration, current use of medication for RA and other medical conditions, and comorbidities. For assessing current medication and comorbidities, we used chart review. RA medication was categorized into synthetic disease modifying drugs (sDMARDs), biological disease modifying drugs (bDMARDs), glucocorticoids, and nonsteroidal antirheumatic drugs (NSAIDs)/other pain killers. Comorbidities were summed up to a total number of conditions.

Pain intensity was assessed via a visual analogue scale (VAS) [29]. Disease activity was measured using the Clinical Disease Activity Index (CDAI), a validated and widely used index [30] that includes the assessment of tender joints, swollen joints and overall disease activity and scores from 0-76 points. Laboratory assessments included C-reactive protein (CRP; mg/dl), tumour necrosis factor-alpha (TNF-*α*; pg/ml), and interleukin-6 (IL-6; pg/ml) to assess the current inflammatory profile of the patients. Blood samples were taken in the morning between 8 am and 11 am.

The functional status of activities of daily living was assessed by the German version of the Health Assessment Questionnaire Disability Index (HAQ-DI), which included 20 questions within 8 categories: dressing, rising, eating, walking, grooming, reaching, gripping, and performing errands. The overall disability index is a value between 0 (no functional disability) and 3 (severe functional disability), representing an average score across the domains [31].

*Lower extremity muscle strength *was assessed by measuring knee extensor strength and lower extremity function. Knee extensor strength was evaluated using an isometric dynamometer that measures the maximum strength of the knee extensor in a standardised procedure [32]. During the test, patients were seated upright with 90° flexion in the hips. A load cell (Chatillon, Ametek Inc®) was mounted on the ankle via a length-adjustable cord. Patients were instructed to perform one maximal voluntary contraction, and strength was assessed three times for each leg, with a two-minute break between measurements. The highest value for each leg, presented in kilogram strength (kg), was taken for statistical analyses. Lower extremity function was assessed using the short physical performance battery (SPPB), a group of measures including tests for gait speed, chair stand, and balance, with a five-level categorical score. The summary score ranges from 0 (worst performance) to 12 (best performance) [33, 34].

*Hand grip strength *was measured using a portable hydraulic hand dynamometer (Sammons Preston, Rolyan, Bolingbrook, IL, USA). Patients were examined in a standardised position, seated upright with their upper arm adducted, and the elbow flexed at 90° [35]. Three measurements with maximum voluntary strength were taken for each hand, in an alternate order, with a two-minute break between each measurement. The maximum value for each hand was taken for statistical analyses.

### 2.4. Statistics

Differences between gainfully employed patients and those without employment were tested using a* t*-test, Mann-Whitney U-test, Chi-squared test, or Fisher's exact test, as appropriate for the data distribution, type of variable, and group size. Univariate linear regression was used to detect associations between muscle strength, lower extremity function, sociodemographic, disease-related, functional variables, and the outcome parameters. Variables that retained association with employment status and work ability in univariate analysis at a level* p*≤0.2 were included in multivariate analyses. Binary logistic regression was then applied to test the odds between hand grip strength, knee extensor strength, lower extremity function (SPPB), and employment status. Multivariate linear regression was applied to assess the effects of hand grip strength, knee extensor strength, and lower extremity function (SPPB) on work ability. A crude model (model I) was stepwise-adjusted for sociodemographic (model II) and disease-related (model III) parameters. For binary logistic regression, exp(*β*) values with a 95% confidence interval were calculated for all included variables, for each step in the respective models, in addition to Nagelkerke's R^2^ as a measure of model fit. For multivariate linear regression, standardised *β*- and* p*-values for all variables, for each step in the model, and explained variance (R^2^) were calculated.

Although gender was not significant in univariate analysis, it remained in the model, since muscle strength is highly dependent on gender [36]. HAQ was not included in the multivariate analysis, due to its mutual relationship with strength and the outcome variables. Disease activity and therapy with NSAIDs/painkillers were not included, due to their multicollinearity with pain intensity. All statistical computations were performed with SPSS version 22.0.2 (IBM Corp, Armonk, NY, USA).

## 3. Results

One hundred and forty patients with seropositive RA visiting the rheumatology outpatient clinic were consecutively screened for eligibility, with a total of 100 patients being included in the present study. To avoid selection bias, we included all eligible patients of working age, with or without current employment. For those with gainful employment at the time of inclusion, the full score of the WAI was calculated, and for those not gainfully employed, only the WAS was assessed. The WAI and WAS showed a high correlation (Spearman correlation coefficient* r*=0.798,* p*≤0.001), indicating good convergent validity between the two measurement instruments. Muscle strength measurements had a high test-retest reliability. The average measure intraclass correlation coefficients with a 95% confidence interval (CI) for right and left knee extensor strength and right and left hand grip strength were 0.97 (0.96; 0.99), 0.99 (0.98; 0.99), 0.99 (0.98; 0.99), and 0.99 (0.98; 0.99), respectively.

### 3.1. Descriptive Characteristics of the Total Population and Stratification by Employment Status

Table 1 shows the sociodemographic, professional, and disease-specific characteristics for the total population and stratification by employment status. The mean age of the patients was 50.5 years old (SD 9.7), and two-thirds were female. Most patients were cohabiting and had at least a secondary school education. In addition, most patients filled in the German version of the questionnaires. The mean disease duration was 9 years, the mean disease activity was low (CDAI=8 points), and the mean pain intensity was 3.6 points on a VAS scale of 1-10 points. Most patients reported no disability and were treated with sDMARDs, and almost half were treated with bDMARDs. More than half suffered from at least one comorbidity and took at least one additional medication. The full WAI and the type of work function could only be assessed for those patients who were employed at the time of study inclusion. The average reported work ability, as measured by the WAI and WAS, was moderate (36.5 and 6.2), with 38% poor, 17% moderate, 36% good, and 9% excellent work ability. Most employed patients had a mixed (physical and mental) type of work and reported a good work ability, as measured by the WAI (37.3 points) and WAS (8 points). The median lower extremity function (SPPB) was good (10.9 points), and the mean grip strength and knee extensor strength were 29.4 kg and 36.4 kg, respectively.

The 41% of patients that were not gainfully employed were significantly older, had a lower level of education, and were more likely to have comorbidities and to take additional medication. Of those without employment, CRP and disability levels were higher. Gainfully employed patients reported a good work ability (8 points) as compared with those without employment, who reported poor work ability (4 points) by means of WAS. Gainfully employed patients had better hand grip and knee extensor strength on both sides. Moreover, lower extremity function was better, as measured by the full score of SPPB and the two subcategories, the chair stands and walking test.

### 3.2. Associations between Muscle Strength, Lower Extremity Function and Employment Status and Work Ability

Tables 2 and 4 show the results of the multivariate regression analyses, with employment status and work ability as dependent variables. The independent variables were those found to be relevant in the univariate analysis (*p*≤0.2), as presented in Tables 1 and 3. In the full adjusted model, patients with better knee extensor strength and physical performance, as well as younger patients, had a significantly better chance of gainful employment. The odds for hand grip strength remained significant when adjusted for sociodemographic, but not for clinical, variables. Better hand grip strength and knee extensor strength together with female gender and lower pain, as well as better lower extremity function (SPPB) together with pain, remained significantly associated with work ability after adjusting for sociodemographic and disease-specific variables.

## 4. Discussion

In the present study, we found an association between employment status, work ability, and parameters of physical fitness. These findings were independent of clinical and socioeconomic parameters.

Despite an improvement in therapeutic management, work disability rates among the RA population are still substantial [9]. There is a broad range of studies regarding work disability and unemployment; however, it must be considered that these outcomes were assessed in different ways and definitions vary among studies, causing difficultly in accurately comparing study results. In the present study,* not being gainfully employed* is defined as not having a paid job at time of study inclusion, whether associated with RA or not; however, in a previous study, unemployment was equated with work disability [37]. In our RA outpatient population, we found that 41% were not gainfully employed and that employment status was not related to disease duration. Looking closer, 56% of the nongainfully employed patients, almost one-quarter of the total study population, had retired early, corresponding to the fraction of work disabled patients in our study.

In a recently published large cohort study, the risk of unemployment was similar in both RA patients and the general population. However, the chance of returning to work following unemployment was significantly lower in the first year after an RA diagnosis and in the subsequent years [8]. Thus, prevention of unemployment appears to be of particular importance in the management of RA patients.

Compared with studies describing good work ability in the healthy population [26], self-reported work ability in our RA population, as measured by the WAS, was moderate (6.2 points), with more than half reporting a poor to moderate work ability. The average WAI in RA patients differs between studies, most likely due to differences in study population characteristics [38, 39]. Here, we show that work ability was significantly higher in the gainfully employed as opposed to unemployed patients. Similar results were observed in an intervention study in Denmark [39], although the WAS levels were slightly lower. In the present study, the WAS scores of gainfully employed RA patients were in accordance with workers who remain in employment despite chronic nonspecific musculoskeletal pain [25].

The major contribution of our study to the field is the observed relationship between grip strength, knee extensor strength, lower extremity function, and work ability and employment status. The association of knee extensor strength and lower extremity function remained significant after adjusting for sociodemographic and disease-related parameters.

In our RA population, hand grip strength [40, 41] and knee extensor strength [42] were markedly lower than the normative values in the general population. A recent study on frailty and physical function in RA patients [43] revealed a lower than average hand grip strength and a similar level of knee extensor strength; however, the study population was slightly older, but the percentage of women was similar. Another study [44] reported similar values for hand grip strength and knee extensor strength in RA patients in remission. Interestingly, this study also showed that, despite improvement in disease control, the relative loss of muscle mass and increased adiposity in RA patients remained, when matched with healthy controls.

Muscle strength as a potential contributor to work ability in RA is amenable to intervention and can therefore be improved. A 2009 Cochrane review [45] suggests that exercise can improve general muscular endurance and strength without detrimental effects on disease activity or pain in RA. Moreover, progressive resistance training with adequate volume appears to be an effective and safe intervention for stimulating muscle growth and reversing cachexia in RA patients [16]. Furthermore, undertaking a specialised exercise program for the hand has been shown to improve hand function, including grip strength [46, 47].

Muscle density has been shown to be strongly associated with disability and lower physical performance in individuals with RA [48]. In our RA population, the average SPPB score was 10.8, and the score of nongainfully employed patients was even lower. This is worrying, since in the older population, limited physical performance of the lower extremities, as measured by an SPPB score lower than 10 points, is predictive of all-cause mortality [49]. Moreover, cognitive function, which is considered a key contributor to work ability [50], was worse in RA patients with a lower SPPB score [51].

In univariate analysis, younger patients with a higher level of education, less pain, lower levels of CRP, the absence of comorbidities, and comedication, and as expected, a higher self-reported work ability, were more likely to be gainfully employed. In multivariate analysis, a younger age remained significantly correlated with a better chance of employment in all models. Better work ability was associated with a younger age, a higher education, longer disease duration, higher disease activity, and more pain. Moreover, female gender and lower pain levels remained independently associated parameters. Functional disability (HAQ-DI) was not included in the multivariate regression but was significantly associated with employment status and work ability in the univariate analysis.

In accordance with our results, a 2006 review summarizing the results of cross-sectional and cohort studies [52] reported that an older age, a lower education level, a higher disability score (HAQ), higher pain levels, and longer disease duration were predictors of work disability. In contrast to our findings, where female gender was associated with better work ability and a higher chance of employment, certain studies have reported that female gender is a risk factor for work disability. It is important to note that, in some studies, it was not specified whether unemployment was related to RA, and different methods were used to assess work disability.

The greatest strength of our study is that it is the first assessment of muscle strength and lower extremity function as potential contributors to work ability and employment status, which greatly contributes to research on work ability among RA patients. Another strength is that we looked at both employment status and work ability; thus, these different concepts can be discussed in parallel. We used standardised objective assessments to quantify grip strength, knee extensor strength, and lower extremity function, and our population represents the typical gender distribution in RA.

There are also some limitations to the present study. The cross-sectional design and relatively small sample size allow us to draw a conclusion from our data about associations, but not about causality. Moreover, the lack of a control group of individuals without RA is a potential limitation. However, literature-reported normative values for the main outcomes and strength parameters are available, and the present study can be considered an approach for generating hypotheses for further interventional approaches or longitudinal studies. Another possible limitation of our methodology is that the population used to create and establish the SPPB value were older adults [34], and the use of this tool is focused on the geriatric population. Nevertheless, since the implementation of SPPB, it has been used in different populations and illnesses in the community dwelling setting [49], including RA [48, 51]. The WAS inquiry ‘current work ability compared to lifetime best' may have led to an underestimation of work ability in older patients, since the disease onset could have been at an older age than that in the younger patients. It is possible that the pain-related noncompliance of patients during physical measurements led to a certain degree of bias in the results; however, multivariate analyses were adjusted for pain levels on the day of the physical tests.

## 5. Conclusions

Work disability occurs early in RA [3]; thus, the WAS should be considered more frequently as an easily applicable outcome measure, since staying at work in spite of this chronic disease must be an important therapeutic goal for physicians and healthcare coverage. We have identified potentially modifiable factors to improve work ability in RA patients. Although interventional studies with specialised rehabilitation programs are needed, our results indicate that work ability and employment may be promoted by improving pain levels, extremity function, and grip and knee extensor muscle strength. According to current guidelines for the management of RA [53], all individuals with RA should be offered access to physiotherapists and occupational therapists. Since prevention of work disability and enhancement of work ability may be more effective than returning to work following unemployment, it seems useful to include functional muscular training, with a focus on strengthening lower extremities in the treatment of RA patients.

## Figures and Tables

**Table 1:** Characteristics of the study population and univariate comparison of subjects with and without gainful employment.

	**Total population(n=100)**			**Gainfully employed subjects(n=59)**			**Non-gainfully employed subject (n=41)**			***p*-value**
**Variable**	**N**	**Mean (SD)/Median [IQR]**	%	**N**	**Mean (SD)/Median [IQR]**	%	**N**	**Mean(SD)/Median[IQR]**	%	
Female gender	66		66	38		64	28		68.3	0.830
Age – years		53.0 [45.0-57.0]			49.0 [40.0-54.0]			58.0 [51.5-61.0]		**<0.001**
In a relationship	71		71	44		75	27		66	0.340
Education level										**0.013**
Compulsory	18		18	5		8	13		34	
Secondary	71		71	46		78	25		61	
Higher	11		11	8		14	3		7	
Disease duration – months		78.00 [36.5-192]			60.0 [31-120]			78.0 (36.5-192)		0.260
Disease activity – CDAI		8.0 (8.2)			0.8 (7.9)			8.4 (8.8)		0.644
Pain - VAS		3.5 (2.05)			3.3 (1.9)			4.1 (2.2)		**0.056**
Inflammatory marker										
CRP - mg/dl		3.2 [1.1-6.7]			2.6 [0.7-5.0]			3.9 [1.7-8.4]		**0.035**
IL-6 - pg/ml		3.9 [1.98-7.91]			3.7 [1.7-7.9]			4.1 [2.2-8.2]		0.443
TNF-alpha - *µ*g/ml		1.6 [0.56-2.35]			1.3 [0.5-2.4]			1.9 [1.1-2.3]		0.273
Rheumatoid arthritis medication										
sDMARDs	80		80	46		78	34		83	0.617
bDMARDs	44		44	30		50.8	14		34.1	**0.107**
Corticosteroids	17		17	10		16.9	7		17	1.000
NSAIDs/Pain killers	15		15	9		15.3	6		14.6	1.000
Comedication (non-RA)	55		55	25		42.4	30		73.2	**0.001**
Number of comedication		1 [0-3]			0 [0-2]			2 [0-4]		**0.002**
Comorbidities										
Presence of comorbidity	65		65	32		54	33		81	**0.007**
Number of comorbidities		1 [0-2]			1 [0-1]			2.0 [1-3]		**0.005**
Functional Disability (HAQ - DI)		0.5 [0.0-1.0]			0.4 [0.0-1.0]			0.8 [0.1-1.3]		**0.009**
HAQ-DI ≤ 1, no disability	70		70	50			27			**0.067**
HAQ-DI >1, disability	28		28	9			12			
Job demands *∗*										
Predominantly physical	10		17							
Predominantly mental	21		36							
Mixed physical and mental	28		47							
Workability index single item (WAS)		7.00 [4.00-7.00]			8.0 [6.0-9.0]			4 [2.5-7.0]		**<0.001**
Workability index (WAI)*∗*		37.3 [32.4-42.0]								
Muscle strength - kg										
Hand grip strength		29.4 (14.4)			32.1(14.9)			25.5 (12.9)		**0.024**
Knee extensor strength		36.4 (16.8)			41.5(17.1)			29.2 (13.6)		**<0.001**
Lower extremity function - (SPPB) total		10.8 (1.9)			11.3 (1.5)			10.1 (2.2)		**0.003 **
Chair stands		3.2 (1.2)			3.6 (0.9)			2.7 (1.3)		**0.010**
Balance test		3.7 (0.7)			3.8 (0.6)			3.6 (0.9)		0.262
Walking test		3.9 (0.5)			3.9 (0.3)			3.8 (0.7)		**0.030**

SD=standard deviation, IQR=interquartile range, CDAI=clinical disease activity index, VAS=visual analogue scale, CRP=C-reactive protein, IL-6=interleukin-6, TNF-alpha=tumor necrosis factor-alpha, s/b DMARD=synthetic/biological disease-modifying antirheumatic drug, WAI=workability index, WAS=single item workability index, SPPB=short physical performance battery, and HAQ-DI=health assessment questionnaire-disability index.

*∗* Assessed for gainfully employed patients only.

**Table 2:** Associations between grip strength, knee extensor strength, lower extremity function, and employment status (dependent variable: gainfully employed).

	Employment status (gainfully employed)									
		Handgrip strength (HGS)			Knee extensor strength (KES)			Short physical performance battery score (SPPB)		
Model	Variable	R^2^	OR	95% CI	R^2^	OR	95% CI	R^2^	OR	95% CI
I	HGS/KES/SPPB^a^	0.09	1.04*∗*	1.01-1.07	0.22	1.06*∗∗*	1.03-1.10	0.14	1.49*∗*	1.14-1.94
II^b^	HGS/KES/SPPB^a^	0.38	1.05*∗*	1.00-1.09	0.44	1.06*∗*	1.02-1.11	0.41	1.46*∗*	1.07-2.00
	Age - years		0.88*∗∗*	0.83-0.95		0.89*∗∗*	0.83-0.96		0.89*∗∗*	0.83-0.95
	Gender: male		-	Reference		- -	Reference		- -	Reference
	female		1.15	0.32-4.16		1.53	0.34-3.96		0.41	0.14-1.23
	Education: compulsory		- -	Reference		- -	Reference		- -	Reference
	secondary		4.12*∗*	1.08-15.71		3.88*∗*	1.01-14.88		4.81*∗*	1.25-18.55
	higher		3.52	0.49-25.93		3.91	0.48-31.66		2.87	0.37-21.87
III^c^	HGS/KES/SPPB^a^	0.38	1.05	0.99-1.12	0.48	1.07*∗*	1.02-1.13	0.48	1.71*∗*	1.18-2.49
	Age - years		0.89*∗*	0.83-0.96		0.90*∗*	0.83-0.98		0.89*∗*	0.82-0.96
	Gender: male		- -	Reference		-	Reference		- -	Reference
	female		1.26	0.30-5.32		1.09	0.29-4.04		0.29	0.08-1.00
	Education: compulsory		- -	Reference		- -	Reference		- -	Reference
	secondary		4.32	1.00-18.57		4.09	0.97-17.26		6.59*∗*	1.42-30.64
	higher		4.81	0.48-47.70		6.05	0.54-68.54		4.97	0.46-53.36
	bDMARD therapy - yes vs. no		2.46	0.85-7.13		2.32	0.78-6.94		2.65	0.88-7.98
	Comedication - yes vs. no		0.67	0.11-3.94		0.48	0.08-2.85		0.66	0.11-3.93
	Comorbidity - yes vs. no		0.93	0.15-5.92		1.46	0.21-10.00		0.93	0.14-6.11
	Pain intensity - yes vs. no		0.92	0.69-1.21		1.00	0.75-1.36		0.96	0.71-1.30
	CRP - mg /dl		1.02	0.98-1.06		1.02	0.99-1.06		1.04	1.00-1.08

Binary logistic regression analysis. OR=odds ratios, HGS=handgrip strength, KES=knee extensor strength, SPPB=short physical performance battery, bDMARDs=biological disease modifying antirheumatic drug, and CRP=C-reactive protein.

*∗p*≤ 0.05; *∗∗p*≤ 0.01; ^a^per kg HGS/KES; per point of the SPPB score; ^b^model II adjusted for sociodemographic variables *p*≤0.02 in univariate analysis; ^c^model adjusted for sociodemographic and clinical variables *p*≤ 0.2 in univariate analysis.

**Table 3:** Univariate analysis of associations between sociodemographic, clinical characteristics, and workability (workability index single item, WAS).

Workability (WAS)		
Variable	*β*	*p*-value
Age - years	- 0.10	**<0.001**
Sex: male	Reference	
female	0.94	**0.108**
Marital status: in a relationship	Reference	
living alone	-0.19	0.757
Education: compulsory	Reference	
secondary	1.26	**0.083**
higher	2.94	**0.005**
Disease duration - months	-0.01	**0.003 **
Disease activity (CDAI) - points	-0.10	**0.003**
Pain (VAS) - points	-0.67	**<0.001**
Functional disability (HAQ) - points	-2.63	**<0.001**
CRP - mg/dl	-0.02	0.275
IL-6 - pg/dl	-0.03	0.291
TNF-alpha - *µ*g/dl	-0.14	0.217
RA therapy: sDMARDs	1.09	**0.111**
bDMARDs	- 1.52	0.787
Corticosteroids	0.32	0.660
NSAIDs/Pain killers	- 1.02	**0.185**
Comedication	-1.81	**0.001**
Comorbidity	-1.50	**0.009**
Muscle strength: hand grip - kg	0.05	**0.012**
Knee extensor - kg	0.06	**<0.001**
Lower Extremity function (SPPB) - points	0.81	**<0.001**

*β*=Beta coefficient, CDAI=clinical disease activity index, VAS=visual analogue scale, CRP=C-reactive protein, IL-6=interleukin-6, TNF-alpha=tumor necrosis factor-alpha, s/b DMARD=synthetic/biological disease-modifying antirheumatic drug, RA=rheumatoid arthritis, and SPPB=short physical performance battery.

**Table 4:** Associations between grip strength, knee extensor strength, lower extremity function, and workability (workability index single item, WAS).

	Workability (WAS)									
		Handgrip strength (HGS)			Knee extensor strength (KES)			Short physical performance battery score (SPPB)		
Model	Variable	R^2^	*β*	*p*-value	R^2^	*β*	*p*-value	R^2^	*β*	*p*-value
I	HGS/KES/SPPB^a^	0.06	0.25	**0.012**	0.17	0.39	**<0.001**	0.33	0.57	**<0.001**
II^b^	HGS/KES/SPPB^a^	0.31	0.49	**<0.001**	0.33	0.52	**<0.001**	0.39	0.50	**<0.001**
	Age - years		-0.21	**0.024**		-0.14	0.132		-0.19	**0.040**
	Gender (female)		0.39	**0.001**		0.35	**0.001**		0.05	0.520
	Education (higher)		0.17	0.064		0.17	0.054		0.14	0.118
III^c^	HGS/KES/SPPB^a^	0.45	0.25	**0.039**	0.45	0.36	**0.001**	0.51	0.35	**<0.001**
	Age - years		-0.15	0.107		-0.09	0.319		-0.14	0.132
	Gender (female)		0.24	**0.032**		0.26	**0.006**		0.06	0.469
	Education (higher)		0.06	0.471		0.08	0.328		0.06	0.505
	Disease duration - months		-0.13	0.189		-0.74	0.391		-0.06	0.511
	sDMARD therapy		-0.07	0.457		-0.10	0.239		0.08	0.317
	Comedication		-0.17	0.193		-0.21	0.104		-0.16	0.217
	Comorbidity		0.06	0.651		0.07	0.589		0.05	0.710
	Pain (VAS) - points		-0.36	**< 0.001**		-0.34	**< 0.001**		-0.39	**<0.001**

Linear regression analysis. *β*=standardised beta coefficient, HGS=handgrip strength, KES=knee extensor strength, SPPB=short physical performance battery, and s/b DMARD=synthetic/biological disease-modifying antirheumatic drug.

^a^per kg HGS/KES; per point of the SPPB score; ^b^model II adjusted for sociodemographic variables *p*≤0.02 in univariate analysis; ^c^model adjusted for sociodemographic and clinical variables *p*≤ 0.2 in univariate analysis.

## Data Availability

The SPSS Database used to support the findings of this study is available from the corresponding author upon request.

## References

[1] Cross M., Smith E., Hoy D. (2014). The global burden of rheumatoid arthritis: Estimates from the Global Burden of Disease 2010 study. *Annals of the Rheumatic Diseases*.

[2] Yelin E., Callahan L. F. (1995). Special article the economic cost and social and psychological impact of musculoskeletal conditions. *Arthritis & Rheumatism*.

[3] Backman C. L. (2004). Employment and work disability in rheumatoid arthritis. *Current Opinion in Rheumatology*.

[4] Sokka T., Kautiainen H., Pincus T., Verstappen SM., Aggarwal A., Alten R. (2010). Work disability remains a major problem in rheumatoid arthritis in the: data from 32 countries in the QUEST-RA study. *Arthritis Research & Therapy*.

[5] Hansen S. M., Hetland M. L., Pedersen J., Ostergaard M., Rubak T. S., Bjorner J. B. (2016). Effect of rheumatoid arthritis on longterm sickness absence in 1994-2011: A danish cohort study. *The Journal of Rheumatology*.

[6] Allaire S. H. (2001). Update on work disability in rheumatic diseases. *Current Opinion in Rheumatology*.

[7] Sokka T. (2003). Work disability in early rheumatoid arthritis. *Clinical and experimental rheumatology*.

[8] Hansen S. M., Hetland M. L., Pedersen J., Østergaard M., Rubak T. S., Bjorner J. B. (2017). Work ability in rheumatoid arthritis patients: A register study on the prospective risk of exclusion and probability of returning to work. *Rheumatology*.

[9] Verstappen S. M., Bijlsma J. W., Verkleij H. (2004). Overview of work disability in rheumatoid arthritis patients as observed in cross-sectional and longitudinal surveys. *Arthritis & Rheumatology*.

[10] Nikiphorou E., Guh D., Bansback N. (2012). Work disability rates in RA. Results from an inception cohort with 24 years follow-up. *Rheumatology*.

[11] Young A., Dixey J., Kulinskaya E. (2002). Which patients stop working because of rheumatoid arthritis? Results of five years' follow up in 732 patients from the early RA study (ERAS). *Annals of the Rheumatic Diseases*.

[12] Dogu B., Sirzai H., Yilmaz F., Polat B., Kuran B. (2013). Effects of isotonic and isometric hand exercises on pain, hand functions, dexterity and quality of life in women with rheumatoid arthritis. *Rheumatology International*.

[13] Mengshoel A. M., Jokstad K., Bjerkhoel F. (2004). Associations between walking time, quadriceps muscle strength and cardiovascular capacity in patients with rheumatoid arthritis and ankylosing spondylitis. *Clinical Rheumatology*.

[14] Stucki G., Bruhlmann P., Stucki S., Michel B. A. (1998). Isometric muscle strength is an indicator of self-reported physical functional disability in patients with rheumatoid arthritis. *Rheumatology*.

[15] Giles J. T., Bartlett S. J., Andersen R. E., Fontaine K. R., Bathon J. M. (2008). Association of body composition with disability in rheumatoid arthritis: Impact of appendicular fat and lean tissue mass. *Arthritis Care & Research*.

[16] Marcora S. M., Lemmey A. B., Maddison P. J. (2005). Can progressive resistance training reverse cachexia in patients with rheumatoid arthritis? Results of a pilot study. *The Journal of Rheumatology*.

[17] Lemmey A. B., Marcora S. M., Chester K., Wilson S., Casanova F., Maddison P. J. (2009). Effects of high-intensity resistance training in patients with rheumatoid arthritis: A randomized controlled trial. *Arthritis Care & Research*.

[18] Walsmith J., Abad L., Kehayias J., Roubenoff R. (2004). Tumor Necrosis Factor-*α* Production Is Associated with Less Body Cell Mass in Women with Rheumatoid Arthritis. *The Journal of Rheumatology*.

[19] Allahan L. F., Bloch D. A., Pincus T. (1992). Identification of work disability in rheumatoid arthritis: Physical, radiographic and laboratory variables do not add explanatory power to demographic and functional variables. *Journal of Clinical Epidemiology*.

[20] Lacaille D., Sheps S., Spinelli J. J., Chalmers A., Esdaile J. M. (2004). Identification of modifiable work-related factors that influence the risk of work disability in rheumatoid arthritis. *Arthritis Care & Research*.

[21] Smolander J., Sörensen L., Pekkonen M., Alén M. (2009). Muscle performance, work ability and physical functioning in middle-aged men. *Occupational Medicine *.

[22] Berner C., Erlacher L., Quittan M., Fenzl K. H., Dorner T. E. (2017). Workability and Muscle Strength in Patients With Seropositive Rheumatoid Arthritis: Survey Study Protocol. *Journal of Medical Internet Research*.

[23] Aletaha D., Neogi T., Silman A. J. (2010). 2010 Rheumatoid arthritis classification criteria: an American College of Rheumatology/European League Against Rheumatism collaborative initiative (Annals of the Rheumatic Diseases (2010) 69, (1580–1588)). *Annals of the Rheumatic Diseases*.

[24] De Zwart B. C. H., Frings-Dresen M. H. W., Van Duivenbooden J. C. (2002). Test-retest reliability of the Work Ability Index questionnaire. *Occupational Medicine *.

[25] De Vries H. J., Reneman M. F., Groothoff J. W., Geertzen J. H. B., Brouwer S. (2013). Self-reported work ability and work performance in workers with chronic nonspecific musculoskeletal pain. *Journal of Occupational Rehabilitation*.

[26] El Fassi M., Bocquet V., Majery N., Lair M. L., Couffignal S., Mairiaux P. (2013). Work ability assessment in a worker population: Comparison and determinants of Work Ability Index and Work Ability score. *BMC Public Health*.

[27] Ahlstrom L., Grimby-Ekman A., Hagberg M., Dellve L. (2010). The work ability index and single-item question: Associations with sick leave, symptoms, and health - A prospective study of women on long-term sick leave. *Scandinavian Journal of Work, Environment & Health*.

[28] Gould RI., Järvasialo J., Koskinen., Gould R. I., Järvasialo J., Koskinen J. (2008). Dimensions of work ability: summary and conclusion. *Dimensions of work ability: results of the Health 2000 Survey*.

[29] Hawker G. A., Mian S., Kendzerska T., French M. (2011). Measures of adult pain: Visual Analog Scale for Pain (VAS Pain), Numeric Rating Scale for Pain (NRS Pain), McGill Pain Questionnaire (MPQ), Short-Form McGill Pain Questionnaire (SF-MPQ), Chronic Pain Grade Scale (CPGS), Short Form-36 Bodily Pain Scale (SF-36 BPS), and measure of Intermittent and Constant Osteoarthritis Pain (ICOAP). *Arthritis Care & Research*.

[30] Anderson J., Caplan L., Yazdany J., Robbins ML., Neogi T., Michaud K. (2012). Rheumatoid arthritis disease activity measures: American College of Rheumatology recommendations for use in clinical practice. *Arthritis care & research*.

[31] Fries J. F., Spitz P., Kraines R. G., Holman H. R. (1980). Measurement of patient outcome in arthritis. *Arthritis & Rheumatology*.

[32] Maughan R. J., Watson J. S., Weir J. (1983). Strength and cross‐sectional area of human skeletal muscle. *The Journal of Physiology*.

[33] Guralnik J. M., Simonsick E. M., Ferrucci L. (1994). A short physical performance battery assessing lower extremity function: Association with self-reported disability and prediction of mortality and nursing home admission. *The Journals of Gerontology. Series A, Biological Sciences and Medical Sciences*.

[34] Guralnik J. M., Ferrucci L., Simonsick E. M., Salive M. E., Wallace R. B. (1995). Lower-extremity function in persons over the age of 70 years as a predictor of subsequent disability. *The New England Journal of Medicine*.

[35] Roberts H. C., Denison H. J., Martin H. J. (2011). A review of the measurement of grip strength in clinical and epidemiological studies: towards a standardised approach. *Age and Ageing*.

[36] Douma R. K. W., Soer R., Krijnen W. P., Reneman M., van der Schans C. P. (2014). Reference values for isometric muscle force among workers for the Netherlands: A comparison of reference values. *BMC Sports Science, Medicine and Rehabilitation*.

[37] Citera G., Ficco H. M., Alamino R. S. P. (2015). Work disability is related to the presence of arthritis and not to a specific diagnosis. Results from a large early arthritis cohort in Argentina. *Clinical Rheumatology*.

[38] Raterman H. G., Hoving J. L., Nurmohamed M. T. (2010). Work ability: A new outcome measure in rheumatoid arthritis?. *Scandinavian Journal of Rheumatology*.

[39] Hoving J. L., Bartelds G. M., Sluiter J. K. (2009). Perceived work ability, quality of life, and fatigue in patients with rheumatoid arthritis after a 6month course of TNF inhibitors prospective intervention study and partial economic evaluation. *Scandinavian Journal of Rheumatology*.

[40] Massy-Westropp N. M., Gill T. K., Taylor A. W., Bohannon R. W., Hill C. L. (2011). Hand Grip Strength: Age and gender stratified normative data in a population-based study. *BMC Research Notes*.

[41] Cuesta-Vargas A., Hilgenkamp T. (2015). Reference values of grip strength measured with a Jamar dynamometer in 1526 adults with intellectual disabilities and compared to adults without intellectual disability. *PLoS ONE*.

[42] Strasser E. M., Draskovits T., Praschak M., Quittan M., Graf A. (2013). Association between ultrasound measurements of muscle thickness, pennation angle, echogenicity and skeletal muscle strength in the elderly. *AGE*.

[43] Andrews J. S., Trupin L., Yelin E. H., Hough C. L., Covinsky K. E., Katz P. P. (2017). Frailty and reduced physical function go hand in hand in adults with rheumatoid arthritis: a US observational cohort study. *Clinical Rheumatology*.

[44] Lemmey A. B., Wilkinson T. J., Clayton R. J. (2016). Tight control of disease activity fails to improve body composition or physical function in rheumatoid arthritis patients. *Rheumatology*.

[45] Hurkmans E., van der Giesen F. J., Vliet Vlieland T. P., Schoones J., Van den Ende E. C. (2009). Dynamic exercise programs (aerobic capacity and/or muscle strength training) in patients with rheumatoid arthritis.. *Cochrane Database of Systematic Reviews (Online)*.

[46] Mathieux R., Marotte H., Battistini L., Sarrazin A., Berthier M., Miossec P. (2009). Early occupational therapy programme increases hand grip strength at 3 months: Results from a randomised, blind, controlled study in early rheumatoid arthritis. *Annals of the Rheumatic Diseases*.

[47] Williamson E., McConkey C., Heine P., Dosanjh S., Williams M., Lamb S. E. (2017). Hand exercises for patients with rheumatoid arthritis: An extended follow-up of the SARAH randomised controlled trial. *BMJ Open*.

[48] Kramer H. R., Fontaine K. R., Bathon J. M., Giles J. T. (2012). Muscle density in rheumatoid arthritis: Associations with disease features and functional outcomes. *Arthritis & Rheumatology*.

[49] Pavasini R., Guralnik J., Brown J. C. (2016). Short Physical Performance Battery and all-cause mortality: Systematic review and meta-analysis. *BMC Medicine*.

[50] Ihle A., Borella E., Rahnfeld M. (2015). The role of cognitive resources for subjective work ability and health in nursing. *European Journal of Ageing*.

[51] Shin S. Y., Julian L., Katz P. (2013). The relationship between cognitive function and physical function in rheumatoid arthritis. *The Journal of Rheumatology*.

[52] Burton W., Morrison A., Maclean R., Ruderman E. (2006). Systematic review of studies of productivity loss due to rheumatoid arthritis. *Occupational Medicine *.

[53] Rheumatoid Arthritis: National Clinical Guideline for Management and Treatment in Adults.

